# Methodology for the Implementation of Internal Standard to Laser-Induced Breakdown Spectroscopy Analysis of Soft Tissues

**DOI:** 10.3390/s21030900

**Published:** 2021-01-29

**Authors:** Anna Šindelářová, Pavel Pořízka, Pavlína Modlitbová, Lucie Vrlíková, Kateřina Kiss, Milan Kaška, David Prochazka, Jakub Vrábel, Marcela Buchtová, Jozef Kaiser

**Affiliations:** 1 Central European Institute of Technology (CEITEC), Brno University of Technology, Purkyňova 123, 612 00 Brno, Czech Republic; anna.sindelarova@ceitec.vutbr.cz (A.Š.); Pavlina.Skarkova@ceitec.vutbr.cz (P.M.); david.prochazka@ceitec.vutbr.cz (D.P.); jakub.vrabel@ceitec.vutbr.cz (J.V.); jozef.kaiser@ceitec.vutbr.cz (J.K.); 2 Faculty of Mechanical Engineering (FME), Brno University of Technology, Technická 2896, 616 69 Brno, Czech Republic; 3 Institute of Animal Physiology and Genetics, Czech Academy of Sciences, Veveří 97, 602 00 Brno, Czech Republic; vrlikova@iach.cz (L.V.); buchtova@iach.cz (M.B.); 4 Third Faculty of Medicine, Charles University, Ruská 2411, 100 00 Praha 10, Czech Republic; katerina.kubickova1@lf3.cuni.cz; 5 Faculty of Medicine in Hradec Kralove, Charles University, Šimkova 870, 500 03 Hradec Králové, Czech Republic; KaskaM@lfhk.cuni.cz; 6 Department of Experimental Biology, Faculty of Science, Masaryk University, Kamenice 5, 625 00 Brno, Czech Republic

**Keywords:** laser-induced breakdown spectroscopy, elemental mapping, soft tissue ablation, zinc, murine kidneys

## Abstract

The improving performance of the laser-induced breakdown spectroscopy (LIBS) triggered its utilization in the challenging topic of soft tissue analysis. Alterations of elemental content within soft tissues are commonly assessed and provide further insights in biological research. However, the laser ablation of soft tissues is a complex issue and demands a priori optimization, which is not straightforward in respect to a typical LIBS experiment. Here, we focus on implementing an internal standard into the LIBS elemental analysis of soft tissue samples. We achieve this by extending routine methodology for optimization of soft tissues analysis with a standard spiking method. This step enables a robust optimization procedure of LIBS experimental settings. Considering the implementation of LIBS analysis to the histological routine, we avoid further alterations of the tissue structure. Therefore, we propose a unique methodology of sample preparation, analysis, and subsequent data treatment, which enables the comparison of signal response from heterogenous matrix for different LIBS parameters. Additionally, a brief step-by-step process of optimization to achieve the highest signal-to-noise ratio (SNR) is described. The quality of laser–tissue interaction is investigated on the basis of the zinc signal response, while selected experimental parameters (e.g., defocus, gate delay, laser energy, and ambient atmosphere) are systematically modified.

## 1. Introduction

Biological tissues contain trace elements that are present in very low concentrations in organisms [1]. Their optimum amount (in microgram to milligram) in living tissues is small but at the same time crucial in order to maintain good health and overall functioning of an organism [2,3]. Trace elements, confirmed to be essential for human organisms, include the following: arsenic, chromium, cobalt, copper, iodine, iron, manganese, molybdenum, nickel, selenium, silicone, vanadium, and zinc. These are known to have an important role in various metabolic processes in a human body [4].

The alteration of trace element metabolism is known to be involved in several pathological processes, including cancer progression. Here, we mainly focus on the zinc detection in soft tissues, as it is one of the most essential metals in human body [3]. Zinc homeostasis is an important factor indicating proper body functioning. Moreover, zinc deficiency and dysregulation may impact body immunity and growth and may even lead to cell apoptosis in severe cases [5]. It has been repeatedly discovered that the zinc concentration in healthy tissue differs from unhealthy organs (prostate [6], kidney [7], breast [8] cancer). These variations in the trace elements accumulation could be effectively used as an indicator of early stages of the disease and as a mean to monitor the progression of malignancy [8].

Several analytical techniques including autometallographical (AMG) imaging [9], X-ray fluorescence (XRF) [8], laser ablation inductively coupled plasma mass spectrometry (LA-ICP-MS) [10], and atomic absorption spectroscopy (AAS) [11] have been previously used for the trace elements sensing in biological samples. LA-ICP-MS provides elemental layout analysis of high sensitivity; its applications were reviewed by Becker et al. [12,13,14,15] and Konz et al. [16]. This method was successfully used to differentiate the tumor areas in paraffin embedded breast tumors [17] and to analyze the distribution of various metals in placental tissue [18]. A new method of imaging of brain tissue of a parkinsonian mouse model with LA-ICP-MS was developed, which resulted in an increased signal-to-noise ratio (SNR), and small but significant changes were detected for tyrosine hydroxylase concentrations [19]. XRF provides results with high sensitivity and resolution and is nondestructive. On the other hand, the detection of light elements (H, C, N, O) is quite difficult, and the quantification is challenging [20,21]. Although each of these methods has its advantages, its application is limited either by the multi-step treatment of samples, the complexity of the required equipment, or the time consuming and costly measurements. Additionally, considering all the above mentioned methods, the elemental layout in tissues can be determined only by using LA-ICP-MS and XRF, however, it struggles with the detection of most non-metals.

Laser-induced breakdown spectroscopy (LIBS) has become a significant method for the elemental composition analysis of any kind and any shape [22,23]. Among other LIBS advantages, the following are relevant for the presented topic: instrumental simplicity, high repetition rate, and possibility of depth-profiling [24,25]. As the speed of LIBS analysis is limited only by detector and laser frequency rates, it can be used for real time elemental imaging, which could be beneficial, for example, during surgery, as was demonstrated previously [26,27,28]. Moreover, it is able to detect most of the elements from the periodic table and gives signal response in real time, which is a significant benefit for a number of applications. This makes LIBS an advanced analytical platform providing the large-scale elemental imaging (whole slide imaging in this case) of heterogeneous sample surfaces. Additionally, LIBS is compatible with the methods listed above enabling higher sensitivity [24,25].

LIBS has a broad range of applications in biomedicine and histopathology, as it is often used for differentiation of healthy and unhealthy tissue in both animals [29] and plants [30]. Moreover, real time identification of various porcine tissues was performed during surgery with high sensitivity and specificity [31,32]. LIBS presents great potential for clinical applications as a means of human tumor diagnosis [28], and its use in dental drilling using lasers was already suggested [33].

Considering elemental mapping, the latest achievements in this field of research were covered by Limbeck et al. and Motto-Ros et al. [34,35]. Laser-induced breakdown spectroscopy was successfully used not only for the elemental analysis in hard tissues, but it has recently become accessible also for the soft tissue assessment. This was demonstrated by using gadolinium based nanoparticles [36] and zwitterionic gold nanoclusters in glioblastoma mouse models [37]. The homogeneity of boron distribution within tumors was assessed by LIBS and further studied by fluorescence imaging [38]. The retention of both La and Ce in various organs of long-term glioblastoma survivors was observed on cryosections with 25 µm resolution [39]. Imaging elemental distribution was studied in the whole mouse kidney in 3D space [40]. Sub-mM sensitivity was achieved during the detection of native elements with artificially added nanoparticles in mouse kidneys [41,42,43,44,45]. Protein biomarkers Leptin and CA 125 (tagged with two-element Fe-Si composite micro-particles) were successfully detected in ovarian cancer tissue [46]. Moreover, the combination of LIBS and LA-ICP-MS improved the accuracy of human tumor analysis and enabled detection of more elements simultaneously [47]. Thorough summaries of LIBS applications and its great potential in imaging of biological samples were recently reviewed by Jolivet et al., Busser et al. and Modlitbová et al. [48,49,50].

In previously mentioned studies, LIBS was used for the detection of selected trace metals within a soft tissue. Despite successful feasibility studies, the sensitivity of LIBS analysis remains a limitation. Such evidence demonstrates the necessity of further research with focus on parameters involved in the laser–tissue interaction in order to improve the detection of large spectrum of trace elements in physiological and pathological tissues.

As mentioned, trace elements are present in the biological tissue in very low concentrations reaching LIBS limits of detection. To be able to detect variations in trace elements concentration and therefore localize any pathological tissue, it is critical to achieve the highest possible analytical sensitivity, which should be provided by additional steps of optimization. The optimization is usually carried out with respect to the element contained in the sample in detectable amounts. However, the content of such an element might vary from sample to sample, which can result in biased signal. Therefore, we suggested implementing the spike method to routine optimization of soft tissue ablation. Its simplicity dwells in the unification of total analyte content to be ablated, and this mitigates any sample to sample variations, making the methodology more robust. In this study, we utilized the spike method where each histological slide was enriched by a monitored content of zinc. In this way, we use samples with standardized analyte content for an extensive optimization.

Moreover, a step-by-step optimization of laser–tissue interaction is presented as the zinc signal response with respect to different analytical parameters. A map of the whole slide was then considered to be a single measurement and a single step of the optimization.

Here, we aim to introduce a guideline for an optimization of LIBS system prior to the analysis of soft tissues. To achieve this goal, we established a methodology focusing on paraffin embedded murine kidneys. Our approach can serve as a guide for the researchers when dealing with the problem of soft tissue analysis and the necessity of robust optimization.

## 2. Materials and Methods

### 2.1. Sample Preparation

Sample preparation significantly influences the performance of a LIBS system, and therefore it is a very important step prior to the analysis itself, especially for soft tissues [51]. Since it is possible to adapt a LIBS system and implement it in a well-established sample pretreatment algorithm, the sample preparation is a flexible step. Homogenizing the sample into a pellet enhances the ablation reproducibility [51]. As we are interested in mapping the elemental layout in a section of soft tissue without affecting the sample matrix, the sample (murine kidney) must stay intact. This is commonly achieved by fixing the tissue section in paraffin, epoxy resin, or freezing the sample. The substrate has a great effect on the measurement, and it strongly affects the laser–matter interaction, as shown in multiple studies [52,53]. This needs to be kept in mind when choosing the appropriate substrate.

Embedding the organ in paraffin is an easy procedure, making a sample more suitable for the whole slide imaging. Since formalin fixation and paraffin embedding (FFPE) is routinely used by pathologists, the implementation of paraffin section imaging in LIBS would open the field to a wide range of applications [54,55]. For these reasons, paraffin embedding technique was applied with the aim of an easy subsequent implementation of LIBS methodology to the histological routine and the future localization of naturally present trace metals in cancerous soft tissues. Here, we used the same routine embedding protocol and applied it on the model of mouse kidneys.

#### 2.1.1. Tissue Processing

Eight to ten weeks old female mice (line originating from the Institute of Cancer Research, ICR) with weight around 40 g were provided by the Masaryk University (Brno, Czech Republic). The zinc concentration in murine kidneys was determined using inductively coupled plasma-optical emission spectrometry (ICP-OES) performed at Masaryk University (Brno, Czech Republic) to be about 19.0 ± 2.0 mg kg^−1^ (Table 1). Mice were sacrificed by cervical dislocation. Kidneys were removed and fixed in 4% formaldehyde overnight in a fridge and processed into paraffin by a standard protocol. The 10 µm thin sections of mouse kidneys were transferred through the water surface (distilled water, 39 °C) on an adhesive glass slide (Superfrost Plus™ Adhesion Microscope Slides, Thermo Fisher Scientific, Waltham, MA, USA). All these procedures were performed at the Czech Academy of Sciences (Brno, Czech Republic). Everything was carried out according to the experimental protocols and rules run by the Laboratory Animal Science Committee of the Institute of Animal Physiology and Genetics (IAPG) (Liběchov, Czech Republic).

It is important not to introduce any zinc throughout the whole sample preparation and make sure that there is no or a negligible amount of zinc in the paraffin or the glass slide used during the sample processing.

#### 2.1.2. Internal Standard

Subsequently, a 10 µL drop of 100 ppm (1.53 mmol l^−1^) Zn solution (99.998%, AN 9069 1N, ASTASOL, ANALYTIKA, Prague, Czech Republic) was added onto the tissue section. The solution was evaporated freely in the open air. Each dried drop had approximately 4 to 5 mm in diameter, thus the surface concentration was 122 to 78 µmol l^−1^ mm^−2^ depending on the exact drop size. This approach then resulted in a series of zinc-enriched sections of murine kidneys. The LIBS map of each section was considered as one measurement.

Finally, BAM 308 (standardized Al alloy with 5.67% of zinc content, Federal Institute for Materials Research and Testing (BAM), Berlin, Germany) was used as a homogeneous reference sample in order to determine the temporal stability of the LIBS system.

### 2.2. Methodological Approach

All tissue samples were prepared by the same procedure; only the parameters of LIBS experiments were adjusted. Standardized samples were prepared with the same known concentration of analyte, having always the same total content of analyte regardless of the map size. By adding a 10 µL drop of Zn solution, we intensified the zinc signal, which facilitated the process of optimization and search for the best experimental settings.

This novel methodological approach overcomes the issue with differences in sizes of individual tissue sections. This methodology enables us to compare individual experiments; a LIBS map of one drop is considered as one measurement, because there is the same content of zinc in each of them. Even though it is not homogenously and identically distributed, the average concentration of analyte in one drop is always the same. Therefore, average values of SNR in different drops should be similar and can be compared among themselves.

### 2.3. Experimental Set-Up

All measurements were performed using the LIBS Discovery instrument, developed at the Central European Institute of Technology, Brno University of Technology (Brno, Czech Republic). The experimental apparatus for LIBS analysis consisted of a Q-switched Nd:YAG laser Quantel CFR Ultra (France; 532 nm, 10 ns, 20 Hz). The laser beam was focused on the sample surface by the triplet lens (Sill Optics, Wendelstein, Germany) with a focal length of 24.5 mm. Plasma emission was collected by wide-angle optics and transferred through an optical fiber to the entrance slit of a Czerny–Turner spectrometer (SR-500i-B2-R, Andor, Northern Ireland) equipped with a grating of 1200 lines per mm and 50 microns entrance slit. Plasma emission was obtained using a gated sCMOS detector (iSTAR-sCMOS-18F-E3, Andor, Northern Ireland). The gate width was set at 50 µs and the gain at 4000. Several experimental parameters were adjusted during the optimization of the detection protocol (Table 2).

As for the elements that were expected to be the most common in the sample (zinc in the drop: Zn I 330.26 nm and Zn I 334.50 nm, calcium in the kidney: Ca II 317.96 nm), central wavelength was fixed at 330 nm with a spectral range from 317 nm to 343 nm. To recognize and assign spectral lines from the acquired spectra to particular elements, National Institute of Standards and Technology (NIST) database [56] was applied.

### 2.4. LIBS Mapping

The whole kidney slice was mapped with a computer-controlled stage. Applying single pulse analysis, spectra were acquired for each sampling position in a matrix with a 100 µm spacing on both axes. The size and the orientation of each murine kidney slice on the microscope slide were different, therefore, the size of the measurement matrix was specific for each sample. A typical map size reached from 100 × 70 to 100 × 100 spots, giving approximately 7000 to 10,000 spectra. Considering a 20 Hz repetition rate and the sample positioning, one sample was measured in approximately 15 min. All the LIBS measurements of mouse kidneys were carried out with gas purge (air or argon) and extraction in order to prevent clogging the lens and the pre-ablations.

### 2.5. Spectra Assessment and Filtering

After each measurement, the background was corrected using moving minimum [57]. The size of the minimum window was set at 50 points and the smoothing window at 30 points. Subsequently, the signal-to-noise ratio for selected peak and noise range was calculated. The signal was defined as the maximum intensity of inspected spectral line in the selected range, and the noise was determined as a standard deviation of all points in the background range. If the SNR value was smaller than five, then these spectra were assumed to lack the zinc content and therefore were filtered out. All spectra with a SNR value higher than five were considered as a part of the analyte drop and a part of the optimization process. This threshold value was established empirically by testing what value reported the most robust results and by inspecting the omitted spectra. The threshold for SNR was the same for all measurements.

## 3. Results and Discussion

### 3.1. The Typical LIBS Spectrum of a Mouse Kidney

**Figure 1:**
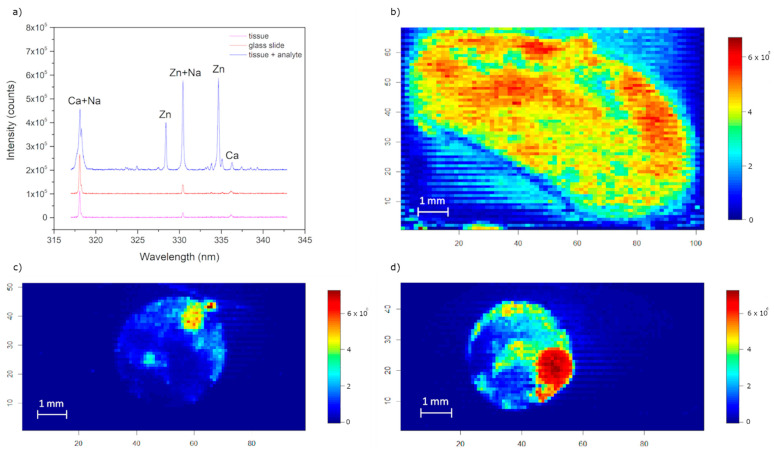
(**a**) Typical laser-induced breakdown spectroscopy (LIBS) spectrum of three different regions in a sample: kidney tissue—magenta color, glass slide with paraffin—red color, zinc drop on the mouse kidney—blue color. Spectra were rearranged vertically for a better clarity. False color map of: (**b**) calcium line Ca I 336.19 nm in kidney tissue; (**c**) zinc drop on soft tissue (Zn I 334.5 nm); (**d**) zinc drop on soft tissue with previously optimized parameters (Zn I 334.5 nm).

The typical LIBS spectrum was acquired by a single shot analysis with the wavelengths ranging from 317 nm to 343 nm (Figure 1a). The identified spectral lines of elements, Zn (drop) and Ca (kidney tissue), are marked. During the measurement, it was observed that the calcium line Ca II 317.93 nm and Zn I 330.26 nm correlated with an element present in the microscope slide. It was most likely sodium, which itself can be seen in at 318 nm, resp. 330 nm (Figure 1a). For this reason, we supposed that the ablation partially took place also in the glass slide. This phenomenon should be minimized and taken into account during the whole optimization process. As a result, considering also a higher Einstein coefficient (Table 3), only the zinc line Zn I 334.50 nm was further deemed as relevant for the zinc sensing and map construction. Analysis of spectra clearly showed that zinc could only be detected in the analyte drop and was not present in traceable amounts in the kidney or in the glass slide (Figure 1a).

### 3.2. Stability of Measurement

**Figure 2:**
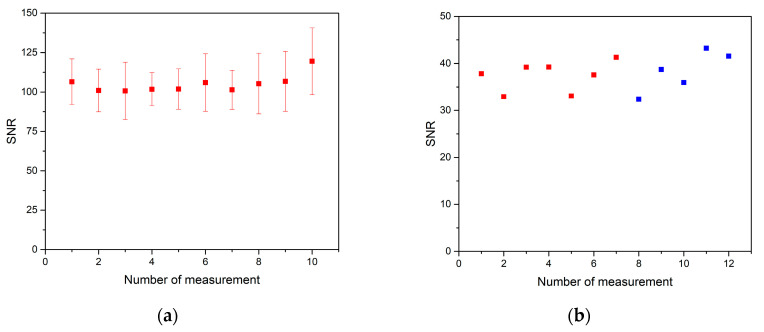
(**a**) The average signal-to-noise ratio of 10 consecutive measurements of BAM 308. Each measurement is a 5 × 5 map, where one spectrum was accumulated from five pulses in one spot. Signal-to-noise ratio (SNR) was calculated in each spectrum as the ratio of Zn I 334.50 nm intensity and respective background in the proximity of analytical line. Then, each point in the plot was statistically obtained as an average of SNR values from 25 spectra. (**b**) The signal-to-noise ratio of zinc solution drop on soft tissue represented by 12 data points, seven red ones (analyzed on 12 July 2019) and five blue ones (analyzed on 23 September 2019). Each SNR in the graph is an average value of SNR from spectra related to one Zn drop. It is not possible to add error lines because SNR is influenced by heterogenous distribution of analyte concentration, therefore, only the average values can be compared.

Before further analysis of the soft tissue itself, we measured a standardized alloy with a high zinc content (BAM 308) homogeneously distributed in its matrix. This was performed to assess the stability of the LIBS system and obtain the analyte signal. Stability was determined as the relative standard deviation (RSD) of repeated measurements [58]. The zinc line Zn I 334.50 nm was considered as previously stated. The signal-to-noise ratio was calculated as a ratio of Zn I 334.50 nm to its respective background. SNR for 10 consecutive measurements (Figure 2a) and the stability of zinc signal from BAM 308 was established to be about 5%, which is reasonable for a typical LIBS experiment conducted on a homogenous metal sample.

The signal-to-noise ratio for zinc solution drop on soft tissue was also assessed (Figure 2b). The latter case was achieved by a consecutive analysis of 12 tissue slices, where seven sections were analyzed on 12 July 2019, and five were analyzed on 23 September 2019. Each point represented an average of SNRs from all spectra related to the Zn drop on individual soft tissue sections. The stability of zinc in the solution drop on soft tissue was about 9%. As it was expected, the presence of zinc in the form of a drop on heterogenous tissue negatively affected the stability of the signal. This fact should be further considered when evaluating results of our study. However, soft tissue sections were also measured on two separate occasions within two months with the same experimental parameters. From the results, we assume this measurement was stable in time and thus repeatable.

### 3.3. Optimization of Experimental Settings

In the beginning of the optimization, a suitable experimental setting was estimated. The laser pulse energy was set at 22 mJ, the gate delay was 500 ns, and the measurements were performed with running air purge in air atmosphere. This setting was proposed based on our experience and current knowledge in this field. The proposed order of the optimization was defocus, then gate delay, laser energy, and lastly gas purge and atmosphere. The step size was maintained constant at 100 microns. However, this is one of the parameters that is necessary to optimize, especially during mapping, in order to achieve the best possible tradeoff between the spatial resolution and the obtained signal. Subsequently, the best result for defocus was fixed, and the gate delay was optimized. The same process was repeated for all the chosen parameters.

The optimization is an iterative process, and individual steps should be repeated several times. However, here, we focused namely on the methodology of constructing the internal standard itself.

#### 3.3.1. Dependence on Ablation Lens Focus

**Figure 3:**
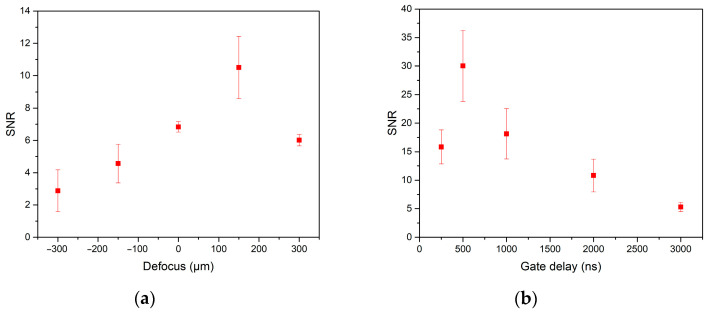
(**a**) The SNR dependence on defocus for zinc spectral line 334.50 nm. Negative defocus means focusing the collection optics above the sample surface, positive defocus means focusing the collection optics under the sample surface, zero stands for the sample surface. (**b**) The SNR dependence on gate delay.

Defocus was adjusted to determine the optimum distance of the sample from the collection optics. The measurements were carried out in a range from 300 μm focus below the sample surface to 300 μm focused above the sample surface with a step of 150 μm. The SNR dependence on defocus was observed (Figure 3a). It was determined that signal-to-noise ratio was the highest when the collection optics were focused 150 μm under the sample surface. The crater diameter during this setting was 50 μm.

The defocus may seem to be excessive in respect to the tissue thickness. However, the ablation does not take place solely in the focus of the laser but also in the place along the laser beam propagation where the irradiance reaches the threshold of a material/medium. This also happens when the focus is below the sample surface. The ablation still takes place on the sample surface, however, with a lower irradiance when ablating larger surface area and with more mass ablated that leads to larger plasma and simultaneously to the increase in SNR. This phenomenon demands further investigation and optimization of energy per spot area (fluence), which will be interesting to follow in the future.

#### 3.3.2. Dependence on Gate Delay

Gate delay stands for the length of time between the laser pulse and the spectrometer detector turning on. The impact of gate delay in the range from 250 ns to 3000 ns was examined. The optimum gate delay was determined to be 500 ns, as it revealed the highest signal-to-noise ratio (Figure 3b). This is a typical result of the LIBS analysis, and it was also demonstrated for the case of soft tissue samples.

#### 3.3.3. Dependence on Laser Pulse Energy

**Figure 4:**
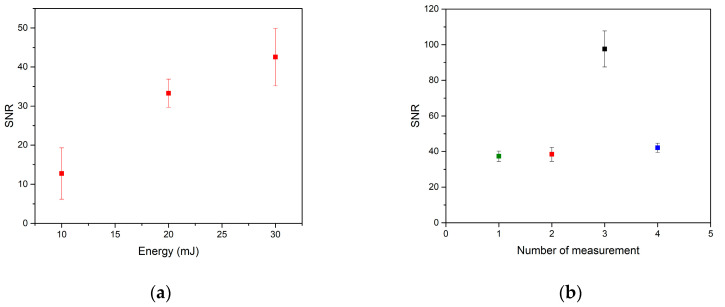
(**a**) SNR dependence on energy for zinc spectral line 334.50 nm; (**b**) SNR dependence on ambient atmosphere (green—analyzed in air purge on 12 July 2019, red—analyzed in air purge on 23 September 2019, black—argon purge, blue—argon atmosphere).

The signal-to-noise dependence on laser energy for 10 to 30 mJ with a step of 10 mJ was studied. It was apparent that the higher the energy of the laser pulse was, the higher the ablated amount of material was and, in turn, the intensity of the analytical line (Figure 4a). However, it must be remembered that the higher the energy is, the larger the craters are and, in turn, the lower the spatial resolution of the LIBS imaging will be. The trade-off between sensitivity and resolution must be established for each sample before further analyses are pursued.

#### 3.3.4. Dependence on Atmosphere

For further analysis, the influence of argon on the analyte detection was investigated. In case of the argon atmosphere, the ablation chamber was evacuated, then it was filled with argon, and the whole process was repeated one more time. Considering argon purge, the experiments were carried out in the ambient atmosphere, but the purge gas was exchanged for argon instead of air.

The effect of atmosphere and argon purge on SNR was evaluated and compared to results with air purge (Figure 4b). The highest signal-to-noise ratio was found using argon purge, being more than two times higher than the rest of the measurements. SNR for the argon atmosphere and SNR for the air purge measurements were approximately the same. The best stability of measurement was found during the experiment with the argon atmosphere, but the improvement compared to air purge measurements was only marginal. For future measurements, analysis in the argon atmosphere using argon purge should offer the best results for both SNR and stability of the measurement.

The reason for significantly better results with argon purge could be a high excitation energy of argon particles (more than 20 eV) compared to commonly present elements in the air (O_2_, H_2_). In the air atmosphere, the excited particles from tissue material would transmit their energy to oxygen and hydrogen particles, leading to a secondary ablation. The signal response would be hence influenced by those elements, and the overall intensity would be lower. During the ablation in the argon atmosphere, the excited particles from tissue cannot transmit their energy to argon particles. This leads to a higher effectivity of ablation of soft tissue and to an increase in signal intensity.

### 3.4. Substrate Effect

**Figure 5:**
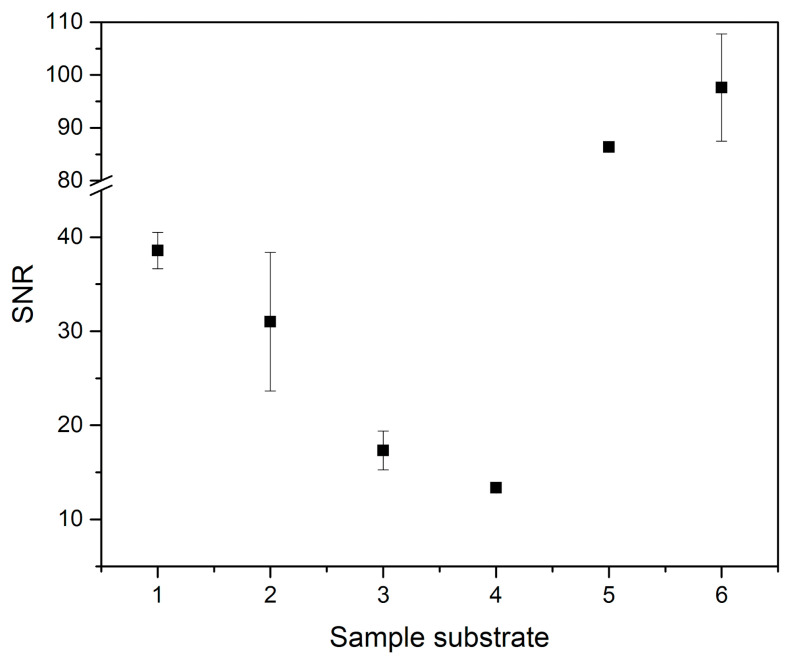
The dependence of signal-to-noise ratio of zinc drops on the type of sample substrate: 1—BAM 310, 2—radish (Raphanus Sativus L.), 3—polystyrene, 4—aluminum foil, 5—glass slide, 6—mouse kidney slice.

We also assessed the effect of substrate on the measurement of zinc drop to prove the feasibility of our approach. The substrates were the following: aluminum alloy BAM 310 (Al 99.85, Mg 0.1), radish (Raphanus Sativus L.; 5-days old plant; dried, embedded in epoxy resin, and placed on a glass slide), polystyrene, aluminum foil, and glass slide. The results were compared with previous measurements of mouse kidney slices embedded in paraffin and mounted on a glass slide. For each substrate, we made five measurements under the same experimental conditions, which were the result of optimization in the case of murine kidneys (energy 20 mJ, gate delay 0.5 μs, defocus 150 μm under the sample surface, argon purge). The process of adding the zinc drops was identical as for previous measurement (Section 2.1.2), and selected substrates contained no or negligible amounts of zinc. The results were plotted to the graph (Figure 5), and the SNR variations were observed.

Despite all the analyte drops having the exact same concentration, the SNR value significantly varied for each substrate type. The highest SNR value was observed for murine kidney tissue, most likely because the experimental parameters were optimized for this specific substrate. Optimization would need to be done for each kind of substrate separately to achieve the ideal conditions. From these results, we can conclude that the substrate significantly affects the signal.

## 4. Conclusions

In this work, we optimized a methodological approach for utilizing an internal standard during the elemental analysis of soft tissue samples using laser-induced breakdown spectroscopy. Using the internal standard will enable robust optimization of the experimental settings. Preparation of such internal standard with zinc solution as well as subsequent analysis on murine kidneys and data treatment were documented. Due to the varying size of samples, a system of mutual comparison was proposed, considering one drop of zinc solution on murine kidney as one measurement. In addition, a brief step-by-step process of optimization of several experimental parameters to achieve the highest SNR was described.

As the laser–matter interaction is related to individual experimental parameters, such as defocus, gate delay, energy, and gas purge/atmosphere, their impact on the analyte detection was found to be significant in kidney samples. Naturally, many additional parameters could be adjusted apart from those suggested in this paper, and all the experimental parameters need to be correlated to each other as they collectively influence the obtained signal. The optimization is an iterative process, and individual steps should be also repeated several times to confirm obtained data. However, this study focused mainly on the methodology of utilizing the internal standard itself and presented such in a manner that could be easily followed in the future while analyzing different types of samples. The main advantage of our approach is that it is a robust methodology which mitigates the sample to sample variations in the macroelement content while keeping the tissue in its natural form—heterogeneous thin sections. Embedding tissue in paraffin and mounting on a glass slide is often used in histopathology, and implementing the internal standard approach in this field would make LIBS more accessible for standard histopathological analysis.

We hope that this methodology will be helpful for those who are beginning to explore this area of research and also for improving routine detection and localization of trace metals present in soft tissues using LIBS in future applications.

## Figures and Tables

**Table 1:** Concentrations of selected elements in murine kidney measured by inductively coupled plasma-optical emission spectrometry (ICP-OES).

Element	Δc (mg/kg)	Standard Deviation	Relative Standard Deviation (RSD)
P	3346	378	11%
K	3013	213	7%
S	2621	267	10%
Na	1644	161	10%
Mg	229	25	11%
Fe	135	32	24%
Ca	75	9	11%
Zn	19	2	11%

**Table 2:** Defocus, laser energy, and gate delay were changed in given ranges throughout the optimization procedure.

Parameter	Range	Unit
defocus	−300 to 300	µm
laser energy	10 to 30	mJ
gate delay	0.25 to 3	µs

**Table 3:** Table of element lines and their attributes (*λ*—wavelength, *A*_ki_—Einstein coefficient, *E*_i_—energy of lower level, *E*_k_—energy of upper level).

Element	*λ* (nm)	*A*_ki_ (s^−1^)	*E*_i_ (eV)	*E*_k_ (eV)
Ca II	317.96	3.6·10^8^	3.1510	7.0500
Zn I	328.23	9.0·10^7^	4.0060	7.7820
Zn I	330.26	1.2·10^8^	4.0300	7.7830
Zn I	334.50	1.5·10^8^	4.0782	7.7839
Ca I	336.19	2.2·10^7^	1.8989	5.5858

## Data Availability

All the acquired data are presented in the form of graphs in the manuscript. The raw data will be provided on request.
